# Analyzing Themes, Sentiments, and Coping Strategies Regarding Online News Coverage of Depression in Hong Kong: Mixed Methods Study

**DOI:** 10.2196/66696

**Published:** 2025-02-13

**Authors:** Sihui Chen, Cindy Sing Bik Ngai, Cecilia Cheng, Yangna Hu

**Affiliations:** 1 Department of Chinese and Bilingual Studies Hong Kong Polytechnic University Hung Hom, Kowloon China (Hong Kong); 2 Social and Health Psychology Laboratory, Department of Psychology University of Hong Kong Hong Kong China (Hong Kong)

**Keywords:** online news coverage, depression, natural language processing, NLP, latent Dirichlet allocation, LDA, sentiment, coping strategies, content analysis

## Abstract

**Background:**

Depression, a highly prevalent global mental disorder, has prompted significant research concerning its association with social media use and its impact during Hong Kong’s social unrest and COVID-19 pandemic. However, other mainstream media, specifically online news, has been largely overlooked. Despite extensive research conducted in countries, such as the United States, Australia, and Canada, to investigate the latent subthemes, sentiments, and coping strategies portrayed in depression-related news, the landscape in Hong Kong remains unexplored.

**Objective:**

This study aims to uncover the latent subthemes presented in the online news coverage of depression in Hong Kong, examine the sentiment conveyed in the news, and assess whether coping strategies have been provided in the news for individuals experiencing depression.

**Methods:**

This study used natural language processing (NLP) techniques, namely the latent Dirichlet allocation topic modeling and the Valence Aware Dictionary and Sentiment Reasoner (VADER) sentiment analysis, to fulfill the first and second objectives. Coping strategies were rigorously assessed and manually labeled with designated categories by content analysis. The online news was collected from February 2019 to May 2024 from Hong Kong mainstream news websites to examine the latest portrayal of depression, particularly during and after the social unrest and the COVID-19 pandemic.

**Results:**

In total, 2435 news articles were retained for data analysis after the news screening process. A total of 7 subthemes were identified based on the topic modeling results. *Societal system*, *law enforcement*, *global recession*, *lifestyle*, *leisure*, *health issues*, and *US politics* were the latent subthemes. Moreover, the overall news exhibited a slightly positive sentiment. The correlations between the sentiment scores and the latent subthemes indicated that the societal system, law enforcement, health issues, and US politics revealed negative tendencies, while the remainder leaned toward a positive sentiment. The coping strategies for depression were substantially lacking; however, the categories emphasizing *information on skills and resources* and *individual adjustment* to cope with depression emerged as the priority focus.

**Conclusions:**

This pioneering study used a mixed methods approach where NLP was used to investigate latent subthemes and underlying sentiment in online news. Content analysis was also performed to examine available coping strategies. The findings of this research enhance our understanding of how depression is portrayed through online news in Hong Kong and the preferable coping strategies being used to mitigate depression. The potential impact on readers was discussed. Future research is encouraged to address the mentioned implications and limitations, with recommendations to apply advanced NLP techniques to a new mental health issue case or language.

## Introduction

### Background

Depression is known as one of the world’s most common mental disorders, with an estimated 5% of adults experiencing it around the globe [1]. The disorder involves a depressed mood, loss of interest in activities, feeling excessive guilt or tiredness, being disconnected from family and friends, and thoughts about dying or suicide that affect all aspects of life. According to the mental health review report [2] released by the Health Bureau in Hong Kong, approximately 1 in 7 Hong Kongers will experience a mental disorder at any given point in life.

Since the outbreak of civil unrest and the COVID-19 pandemic in Hong Kong, a surging number of studies have emerged to examine the intensity of local people’s depression, particularly among adolescents, older adults, students, and health care workers [3-6]. In particular, these studies have been widely associated with social media use and its effect compared to other digital media use [7-11]. Although the online press is one of the most widely consumed digital media in Hong Kong, existing research on online news coverage of depression has primarily been conducted in the United States, Australia, Canada, and China [12-17]. Studies have rarely explored online news coverage of depression in Hong Kong, based on available research.

The consumption of news coverage on depression can have both beneficial and detrimental effects on its readers. According to the protection and motivation theory, which has been applied in investigating different health-related behaviors, the underpinned threat and coping appraisal shape individuals’ belief of the potential dangers to their lives and the motivation to practice protective behaviors via communication [18,19]. Thereby, positively, news coverage can help to raise awareness, increase understanding of mental disorders, and may facilitate the intention of professional help-seeking behavior [18,20]. Negatively, news coverage can also facilitate misunderstanding of depression among the public, exacerbating the stigma regarding people with mental disorders and leading to undertreatment [12,16,21,22]. The impact of news consumption has been acknowledged, and in depression-related news, diverse subthemes have also been investigated.

According to research on US television news coverage, the primary latent subthemes associated with individual and family-related depression stories include entertainment and celebrity depression, personal experiences, crime, and public policy [23]. Most of the news reports were also found to be largely negative in tone and often occurred with the subthemes of violence and crime [16]. This was likely because negative news can disproportionally grab the readers’ attention, while positive news is perceived as having minimal impact [24]. Such a trend of news coverage is thereby harmful to populations with depression as it may reinforce social stigma and decrease public support for individuals with mental health disorders [16].

Conversely, in other research, which analyzed the contents of Canadian print news, the primary theme was raising awareness with the subthemes of addressing policy issues or lack of resources, military mental health issues, workplace issues, stigma, and so on, followed by the theme of research advances encompassing subthemes of treatment discoveries, etiology or prevention, and personal stories of recovery [25]. These kinds of positive subthemes may bring a positive effect of news exposure, which leads to favorable outcomes on mental health, particularly in circumstances where access to social support is restricted [26]. It is a fact that depression is 1 of the 2 most frequent mental disorder diagnoses In Hong Kong and has continued to deteriorate in recent years [3,27]. Media coverage of depression may encourage positive outcomes such as enhancing social support for individuals with depression or otherwise lead to negative outcomes, such as strengthening the public stigma toward the population with depression and their self-stigma [15,16,22,26]. Investigating the underlying subthemes of online news could aid in comprehending the positive and negative impacts of news experienced by its readers and potential mental health outcomes on populations with depression. However, to the extent of available studies, limited research has investigated the subthemes associated with online news coverage of depression in Hong Kong.

### Research Questions

This study aims to fill this gap by exploring the latent subthemes in the news, which constitutes our first research question (RQ): What are the latent subthemes in the online news coverage of depression in Hong Kong? (RQ1)

In contrast, the sentiment of the news, which can be categorized as positive, negative, or neutral, has a direct effect on the stigmatization or destigmatization of people with depression or mental disorders [28,29]. Compared to print media, online news coverage was found to have a more positive sentiment in Canada and Chile, which helps to raise awareness, increase rehabilitation and recovery, and reduce the stigmatization of populations with depression [29,30]. Conversely, another study in the United Kingdom found that half of the articles about mental health expressed a negative sentiment and were closely connected with subthemes of violence, danger, and criminality, which reinforced stigma toward individuals with depression [31]. Stigmatization of people with depression usually labels them as having bad character, being violent, and blaming their conditions as a personal weakness, consequently, adding more obstacles when they want to seek social support [13,21,25]. People who are experiencing depression may also internalize the stereotypes, leading to diminished self-esteem, reduced quality of life, reluctance to seek help, and an increased likelihood of engaging in suicidal behavior [22,25].

Therefore, the sentiment of online news can play a significant role in reproducing or challenging the stigma around depression [32]. Within the context of Hong Kong, the embedded cultural norms, particularly “face concern,” make people with depression avoid seeking help from mental health professionals to prevent shaming their own families [33]. A study that investigated the experiences of mental health service users comparatively in 2001 and 2017 found no positive attitudinal changes toward people with mental disorders in Hong Kong due to perceived stigmatization [34]. However, reporting of celebrity depression, particularly their stories of overcoming depression, has resulted in positive attitudinal changes in Hong Kong, motivating readers to learn about available treatments for depression [20]. News that expressed more negative sentiment is correlated with increased anxiety, depression, and psychological distress in its readers [35,36]. Given the potential impact of news, it is important to investigate its sentiment to enhance our comprehension of its influence on both populations with depression and the broader audience. As most journalists are unaware that their reporting may have varying impacts [37] and the sentiment of online news was also less studied in the Asian context, analyzing the sentiment could further provide practical suggestions for future news practices in Hong Kong based on solid research findings. Consequently, this study is also interested in exploring how different sentiments were reflected in different latent subthemes, which constitutes our second RQ: What sentiments are reflected in the online news coverage of depression in Hong Kong and are associated with the latent subthemes? (RQ2)

In addition, starting from early research, it is worth noticing that news coverage on mental disorders provided rare coping resources or information for populations with depression [12,15,25,37,38]. However, the provision of coping strategies (ie, information on skills and resources and emotional support) is important to reduce uncertainty about a certain disease and to facilitate the potential of help-seeking behavior [39]. Therefore, news coverage was suggested to leverage its impact and include more information and resources about depression treatments or prevention, as it may help to reduce the individual’s uncertainty about depression, decrease public negative attitudes on depression, and increase public awareness and knowledge of this certain mental health disorder [13,18,40,41]. Therefore, this research also seeks to analyze whether online news coverage of depression provides coping strategies for supporting the population with depression in Hong Kong, which constitutes our third RQ: What kind of coping strategies were provided in the online news coverage of depression in Hong Kong? (RQ3)

## Methods

### Data Selection and Collection

This research aimed to collect depression-related news on Hong Kong mainstream news websites and analyze the collected news using the Natural Language Toolkit (NLTK) embedded in Python. The latent Dirichlet allocation (LDA) topic modeling was first conducted to address RQ1, followed by the Valence Aware Dictionary and Sentiment Reasoner (VADER) sentiment analysis to address RQ2. RQ3 involved manual coding of coping strategies by content analysis. The detailed methodology will be later elaborated in the Data Analysis subsection.

To conduct a comprehensive search and collection of depression-related news in Hong Kong, the *Hong Kong Free Press* (*HKFP*) and the *South China Morning Post* (*SCMP*) were carefully selected based on 3 criteria. First, news websites should be accessible to readers without membership needs to prevent potential news access privacy risks. Second, the news website could retrieve a certain amount of depression-related news (ie, >20) to guarantee adequate data for subsequent analysis. Third, the news websites are published in English. Given that Hong Kong is a multilingual society with diverse ethnicities, English is one of the most used languages and provides a broader reach among diverse reader groups in Hong Kong. Online news websites that do not meet these 3 criteria were excluded.

After selecting the websites, the news was searched by the keywords, “depression” and “depressed” separately in each news website to ensure all related news was included as per the search strategy used in previous research [21,41,42]. Only news articles published between February 2019 and May 2024 were collected during the search process. In February 2019, the introduction of the Fugitive Offenders and Mutual Legal Assistance in Criminal Matters Legislation (Amendment) Bill sparked protests and marches in Hong Kong due to divisive political viewpoints, leading to a significant rise in depression cases, which worsened in 2020 with the outbreak of COVID-19 [6,43,44]. Therefore, the data were aimed to be collected during and after this period to obtain a more updated picture of the media portrayal of depression in Hong Kong. News outside this time range was excluded, and data collection concluded on the author’s final search day.

Eventually, 3648 news stories were collected. Specifically, when searching with the keyword “depression” in the *SCMP*, 2298 (62.99%) news articles were retrieved, and 1040 (28.51%) more news articles were collected by searching with the keyword “depressed.” In the *HKFP*, each keyword retrieved 155 news articles, resulting in a total of 310 (8.5%) news articles being collected. Afterward, all collected news was manually exported to a Microsoft Excel file for screening and excluding duplicated news based on the titles. Meanwhile, nonnews articles, such as opinions and commentary, were excluded. Therefore, of 3338 *SCMP* news articles, 209 (6.26%) duplicated news articles and 846 (25.34%) nonnews articles were excluded, leaving 2283 (68.39%) news articles for subsequent analysis. Furthermore, 158 (51%) duplicated news articles were removed from 310 *HKFP* articles, resulting in 152 (49%) news articles. The dataset thus included 2435 news articles for data analysis.

### Data Preprocessing

Before applying the natural language processing (NLP) techniques for each RQ, each news article was uploaded to Python and created as a news corpus. The corpus was first preprocessed and tokenized into words to ensure better clarity and results of analysis [45]. Tokenization in NLP means converting words or sentences in a text into understandable tokens that a program can work with. After tokenizing the corpus, lemmatization was performed to convert words to their root meaning, such as “works,” “working,” and “worked” to “work.” Stop words were removed by using the NLTK library. Common stop words, such as, “the,” “and,” “in,” and punctuations were removed as they do not provide meaningful information for subsequent data analysis. Afterward, we observed the top 200 words from the corpus and created a stop word list to remove other uninformative words, such as “say,” “year,” and “people.”

### Data Analysis

To uncover the subthemes underneath the online news for RQ1, LDA was first performed on the combined dataset to investigate the “hidden” thematic topics. The distribution of these topics will be then examined in each dataset separately to allow a more nuanced understanding. Specifically, LDA is one of the unsupervised topic modeling techniques and is becoming increasingly popular in communication research due to the ability to quickly identify the thematic structure of large amounts of text and allow more efficient explorative and descriptive analyses [45]. LDA assumed that each document or news was represented as a mixture of topics, and each topic was characterized by a distribution of words [46]. The algorithms started by assigning topics randomly to the document and then computed the distributions of words within a topic, as well as the distribution of topics within a document, then continued to update and iterate until the most suitable match was found [47]. The *genism* package was used in the NLTK to perform LDA analysis.

Meanwhile, after performing LDA analysis, coherence is an essential metric that decides the best number of topics (k) for the designated corpus. It measures the quality of the topics generated by the model and how semantically interpretable the topics are. A coherence range of 0.3 to 0.8 is acceptable [48-50]. To gain a more comprehensive understanding of the intertopic relationships, multidimensional scaling (MDS) is also used to assess and visualize the topic distance between each other based on their word distributions [51]. Specifically, topics exhibiting similarities will be positioned closer to each other on the MDS map, while topics with fewer similarities will be placed farther apart. MDS helps to facilitate the decisions regarding whether certain topics should be merged or excluded based on their similarities or distances. In contrast, the LDAvis tool (set λ=1) assists with inspecting the top keywords or most associated terms under each topic based on their relevance [52]. Relevance refers to the probability of occurrences of a term under a topic and is ranked from top to bottom to aid topic interpretation.

Moreover, sentiment analysis is another NLP technique that enables the analysis of sentiment being expressed in online news to address RQ2 [53,54]. VADER is a lexicon-based sentiment analysis tool that is included in the NLTK and shows high accuracy in news sentiment analysis [55]. In sentiment analysis, each news article was treated as a document, and the polarities of individual words within the document were calculated and summed to categorize each news into positive, negative, or neutral stances by providing a compound sentiment score [28]. The compound sentiment score ranged from –1 to 1, where –1 indicated the most negative sentiment and +1 indicated the most positive sentiment [56].

For RQ3, 2 authors who are doctoral students in language and communication studies coded each piece of news sentence by sentence to see whether the news provided coping strategies for the population with depression. Although NLP techniques can efficiently process large datasets, they may lack the nuance and contextual understanding that human coders could interpret. Manual coding can offer a deeper investigation into the news and provide more robust research findings [39,57]. The coding scheme was adapted from 2 social media studies investigating depression and coping strategies in health crises [12,39]. Specifically, each news was manually read and labeled by the first and fourth author in 2 rounds of coding.

The first round involved coding for the absence or presence of coping strategies in the news. The second round involved coding in the news that presented coping strategies and further labeled them based on the following categories: (1) information on skills and resources, (2) emotional support, (3) individual adjustment, and (4) others. The first and second categories correspond to the framework designed by Ngai et al [39] to assess if the news articles include information on coping with depression and provide supportive resources as well as emotional support. The third category aligns with the coding done by Pan et al [12], which examines whether the news provides self-adjustment strategies to cope with depression without seeking external help and is found to be the most used coping strategy to mitigate depression on social media. The fourth category refers to news that includes other coping strategies that are not covered by the previous 3 categories.

**Figure 1:**
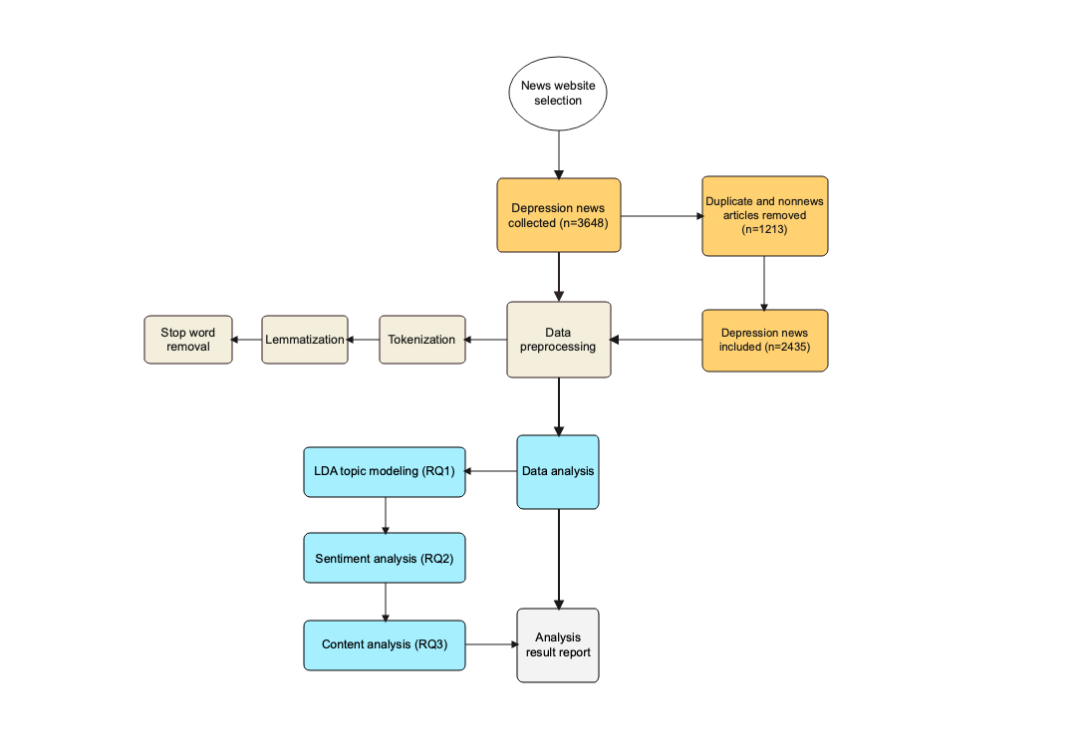
Flowchart of data collection, preprocessing, and analysis. LDA: latent Dirichlet allocation; RQ: research question.

To ensure consistency in the application of the coding, Cohen κ, a statistical measure, was applied to measure the intercoder reliability on 2435 news articles [57]. The κ value ranges from –1 to 1, suggesting that a value of 0.8 in each round is the minimum acceptable intercoder agreement [58]. The flowchart in Figure 1 outlines the overall process of data collection, preprocessing, and analysis.

### Ethical Considerations

This study was conducted in accordance with ethical standards and guidelines. Institutional ethical approval was not sought as the research did not involve interaction with human subjects [59]. The data used in this study were obtained from publicly available resources and were analyzed in a manner that ensured the privacy and confidentiality of any individuals indirectly involved.

## Results

### LDA Topic Modeling

**Figure 2:**
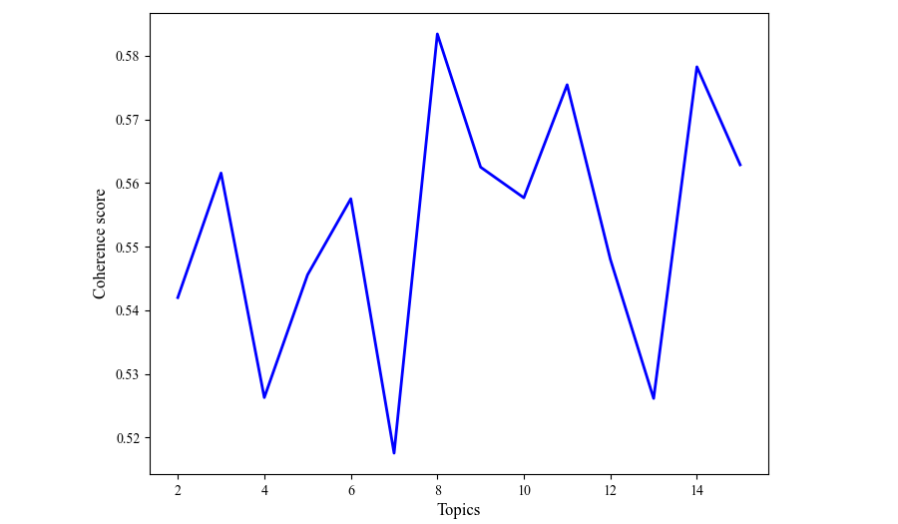
Coherence scores of varied topic numbers.

**Figure 3:**
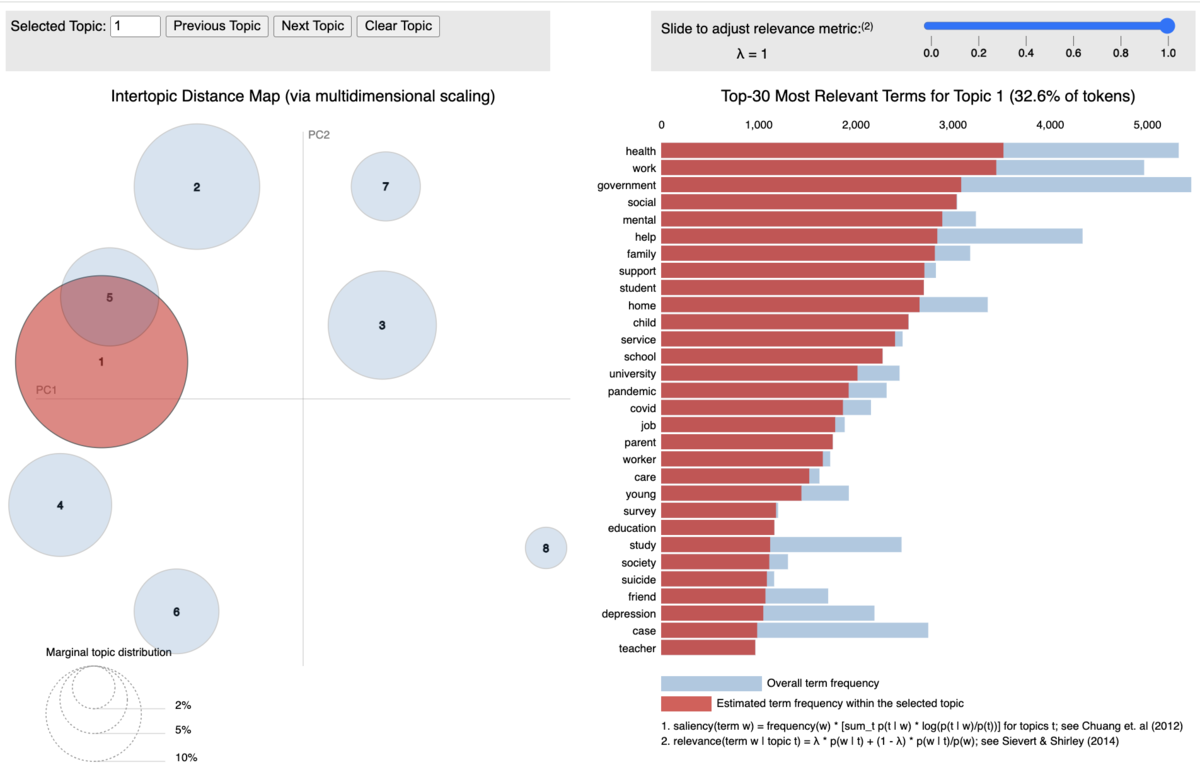
Intertopic distance via multidimensional scaling (MDS) and Top 30 words for topic 1 via LDAvis. PC1: Principal Component Analysis 1; PC2: Principal Component Analysis 2.

After data preprocessing, the LDA topic modeling was first used for RQ1. We examined the coherence scores in different k from 1 to 15 and found the coherence score reached its peak at k=8, where the coherence value equaled 0.5834 (Figure 2). Meanwhile, we visualized the intertopic relationships between the 8 topics by MDS, as illustrated in Figure 3 [51]. The topic keywords under each topic were also presented by LDAvis on the same figure [52]. However, it is worth noticing that LDA-generated topics might not be readily interpretable. Previous research has suggested labeling the topics into meaningful frames to better interpret the LDA-generated topics [60,61]. Having an expert in a related research field to label the frame or review the top documents and top words under each LDA topic are both feasible approaches that have been applied in previous research [45]. Therefore, the third author, who is a public health expert, particularly in the field of social psychology, reviewed each topic, the most relevant news under each topic, and top keywords from LDAvis to label the topic frames, as shown in Table 1.

**Figure 4:**
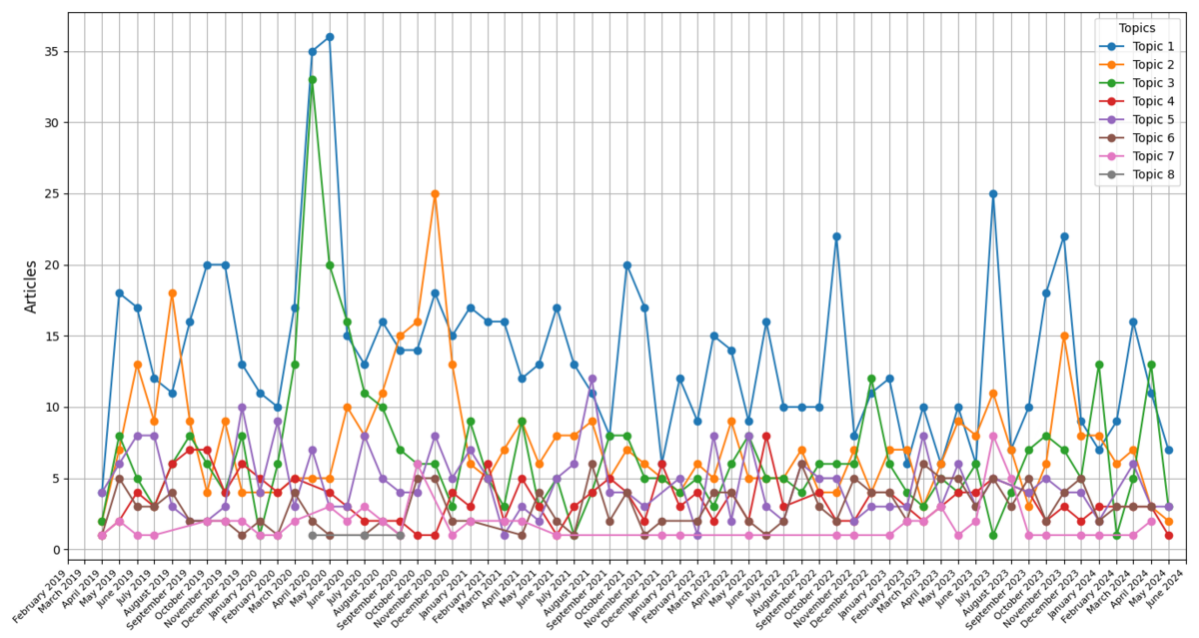
Trend of news articles by topic.

In addition, drawing from observation, topic 3 and topic 8 were merged into one frame as topic 8 only occupied a small share of the news according to MDS, and its underlying top keywords and news were more aligned with the content of topic 3. Subsequently, we examined the LDA topic labels within the *HKFP* dataset and revealed that a significant proportion of the news articles, specifically 74 (48.7%) of the 152 news articles, were allocated to topic 1. This was followed by 59 (38.8%) of the 152 news articles assigned to topic 2. In addition, 10 (6.6%) news articles were labeled under topic 5, while 9 (5.9%) articles were equally assigned to topics 7, 4, and 3. On the basis of the identified topics, we also examined the reporting time of the news under each topic to visualize the overall temporal trend (Figure 4).

**Table 1:** Topic frames, top keywords, and illustrative quotes in Hong Kong depression news.

Topic frames or subthemes	Topics	Top keywords	Illustrative quotes	News portion (N=2435), n (%)
Societal system	Topic 1	health, work, government, social, mental, help, family, support, student, and home	“The number of Hong Kong students with mental health problems shot past 1,400 in the last academic year...with experts and educators blaming the 2019 social unrest and the Covid-19 pandemic...Some feared that the actual number of young people with mental health issues could be much higher...included anxiety, depression, obsessive-compulsive disorder...and eating problems.”	848 (34.83)
Law enforcement	Topic 2	police, law, protest, case, government, officer, china, protester, force, and security	“A real estate agent accused of slashing a man with a knife and assaulting another during an antigovernment protest on Friday has been remanded to a psychiatric facility, after a court heard he had been battling depression for a year.”	458 (18.81)
Global recession	Topic 3	china, market, chinese, unitedstates, company, trade, business, Beijing, government, and bank	“After three years of the pandemic, the market should cope better than before, especially when valuation is so depressed,’ he said. ‘Nevertheless, investors should be prepared for market volatility ahead.’”	412 (16.92)
Global recession	Topic 8	economic, economic, growth, economist, lockdown, virus, recovery, spending, tax, and inflation	“New Zealand suffered its worst economic slump since the Great Depression in the second quarter as a strict nationwide lockdown to combat the coronavirus brought the country to a standstill.”	3 (0.12)
Lifestyle	Topic 4	sleep, help, drink, eat, bed, body, work, stress, gas, and mind	“The genetic study...by the University of Colorado Boulder, the Broad Institute at MIT and the University of Harvard...found that waking just one hour earlier—but getting the same amount of sleep—reduces your risk of major depression by 23 percent.”	196 (8.05)
Leisure	Topic 5	film, work, story, game, sport, woman, video, music, event, and book	“Osaka withdrew from last year’s French Open after being fined and threatened with expulsion for refusing to do media duties, which she said had contributed to the depression she had struggled with for years.”	273 (11.21)
Health issues	Topic 6	patient, health, treatment, study, symptom, doctor, diseases, cancer, drug, and hospital	“This discovery is expected to provide rapid and robust therapies, and most importantly, if the transcorneal electrical stimulation is effective in reducing the symptoms of depression and dementia, then this could be a major breakthrough for patients, families and caregivers.”	169 (6.94)
US politics	Topic 7	election, trump, party, unitestates, candidate, democracy, president, political, vote, and gay	“Fetterman’s dress style, or perhaps lack of style, became his signature on the campaign trail before entering the Senate this year. He also gained sympathy from many after he had to undergo treatment for clinical depression soon after taking office.”	76 (3.12)

### Sentiment Analysis

After uncovering the subthemes in the online news, our next objective was to ascertain the prevailing sentiment conveyed in the news. Sentiment scores were computed for each news using VADER. The findings indicated that the collective sentiment expressed in the news articles leaned toward the positive spectrum. Specifically, 50.51% (1230/2435) of the news articles reflected a positive stance, whereas 49.08% (1195/2435) of the news articles reflected a negative sentiment. Moreover, 10 (0.41%) pieces of news maintained reflected a neutral stance.

**Figure 5:**
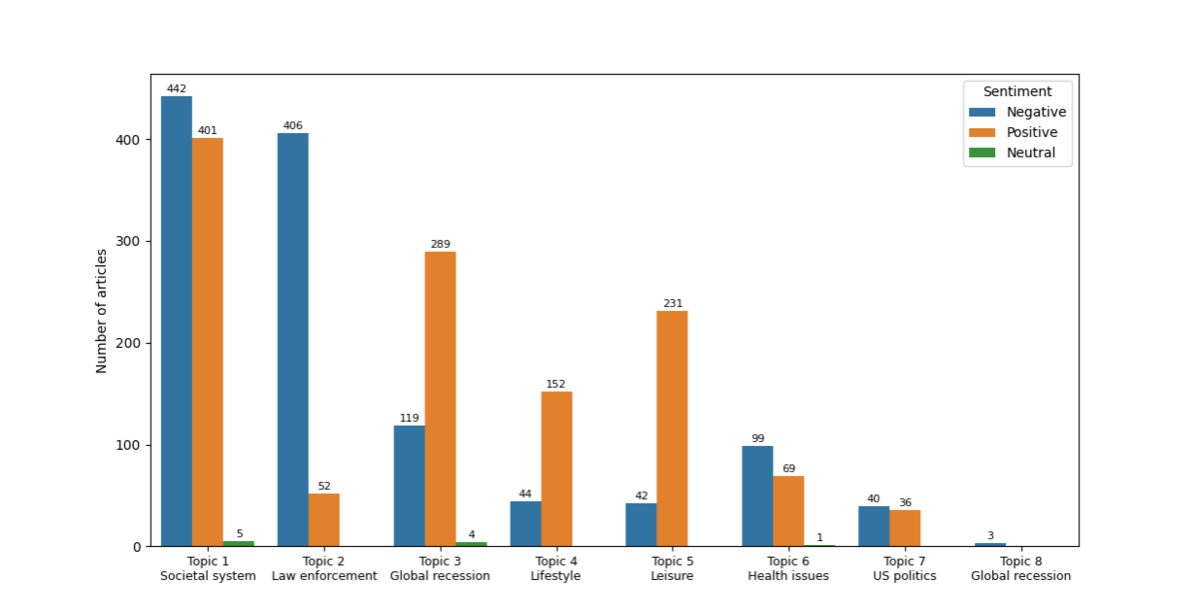
Sentiment distribution by topic.

Consequently, we correlated the sentiment scores in different LDA topics to observe the sentiment variation in response to RQ2 (Figure 5). Notably, topics 1, 2, 6, 7, and 8 are more negatively inclined, while topics 3, 4, and 5 are positively oriented. Therefore, subthemes of the societal system, law enforcement, health issues, and US politics exhibited a predominantly negative sentiment. In contrast, subthemes of the global recession in a combination of topic 3 and topic 8 sentiment results as well as lifestyle and leisure exhibited a more positive sentiment.

### Coping Strategy Analysis

RQ3 focused on examining whether online news has provided depression-related coping strategies. It was found that >76% (1861/2435) of the news did not present coping strategies. After the two coders labeled all the news respectively, the interrater reliability in identifying and categorizing the coping strategies across 2 rounds was assessed using the Cohen κ statistic [58]. All disagreements between the coders were discussed. We used the SPSS (IBM Corp) crosstab function to calculate Cohen κ in both rounds of labeling. In the first round, the Cohen κ value obtained was 0.882, indicating a substantial level of agreement regarding the presence or absence of coping strategies in 2435 news articles. For the second round, we focused only on the news initially labeled as “2” in the first round, in which 501 (20.57%) news articles were consistently annotated by both coders, emphasizing particular coping strategies were presented. The κ value obtained for the second labeling round yielded a value of 0.849, suggesting a firm agreement between coders regarding the specific categories of coping strategies.

Among the news that presented coping strategies, most of the news articles (334/501, 66.7%) provided information on skills and resources to cope with depression. Under this category, local mental health organizations or charities launched diverse mental health programs and provided support hotlines to facilitate quick resource access. This was followed by individual adjustments, including making personal changes, such as adopting a healthier lifestyle through exercise, sleep, and diet. Emotional support was the third preferred coping strategy, including support from friends, family, partners, and pets to help individuals mitigate depression. The least mentioned category includes mental health advice suggested by experts, particularly focusing on parental guidance for the well-being of their children and coverage of past mental health initiatives.

## Discussion

### Principal Findings

This study investigated online news coverage of depression in Hong Kong, discovering prevalent subthemes (RQ1), overall sentiment (RQ2), and coping strategies (RQ3) in the news. In response to RQ1, the topic modeling results identified 8 LDA topics based on the coherence score, and 7 latent subthemes were eventually identified by using MDS, LDAvis, and expert insights. The primary subtheme is “societal system,” accounting for a representation of 34.83% (848/2435) of overall news, followed by “law enforcement” (458/2435, 18.81%), “global recession” (415/2435, 17.04%), “lifestyle” (196/2435, 8.05%), “leisure” (273/2435, 11.21%), “health issues” (169/2435, 6.94%), and “US politics” (76/2435, 3.12%). The overall sentiment of the news was slightly more positive, which aligns with news reported in Canada and Chile [29,30,62]. Among the subthemes, “societal system,” “law enforcement,” “health issues,” and “US politics” exhibited negative tendencies, while others tended to be more positive. The coping strategies were substantially inadequate, in line with previous research results [25,38,40]. However, this study has enhanced comprehension regarding preferred types of coping strategies provided in depression-related news, predominately featuring information on skills and resources and individual adjustment to cope with depression.

On the basis of the sentiment analysis, it becomes apparent that the primary subtheme, focusing on the societal system, exhibited a more negatively inclined sentiment. The subtheme was related to diverse systems in Hong Kong’s society, including the health and social systems, such as family, education, and work. The amount of news by reporting time indicated a peak in reported news, notably during May 2020, April 2020, and July 2023. Therefore, we first examined the news content between April and May 2020 and found that COVID-19 lockdown measures greatly affected Hong Kong, contributing to a more depressed mood [6]. Specifically, the period signifies the second wave of the COVID-19 outbreak [63]. While the first wave in Hong Kong reported relatively few cases, the second wave saw a sharp increase in cases, leading to stricter measures to contain the virus, including school closures, work-from-home policies, and a ban on dine-in services [43]. School closures, as noted in the news, resulted in an increased rate of depression among children and heightened anxiety for parents who had to take care of both their children and the older adults at home. Work-family conflicts also elevated due to the disruption to normal work routine (ie, no office space and blurred working time), which was found to be depressing [8,64]. In July 2023, we continued to examine the news content and found that reported suicide cases surged. Notably, an influential star, Coco Lee, who experienced depression, died by suicide at home, raising more awareness for expanded mental health support services and enhanced accessibility, with support hotlines being frequently highlighted. This phenomenon is supported by previous research, which suggests that news of celebrity suicides is more likely to provide coping strategies, as readers, particularly fans, may become more proactive in seeking treatment for depression and increase their understanding of the condition [18,20].

The second leading subtheme focuses on law enforcement, mainly negative sentiment, and coincides with the subtheme in other news studies [23,42,65]. The amount of reported news peaked during November 2020 and August 2019. In the former month, individual crimes and court cases were reported, particularly centering on an event of a Hong Kong professor murdering his wife, which may have brought great social shock and attention. Notably, depression was often cited as an explanation for the perpetrator’s illegal actions in the news, which may reinforce stigmatizing attitudes and portray people with depression as violent, dangerous, and more likely to commit crimes [13,16,23,31]. The stigmas associated with depression could have been strengthened by the news report, making the public more unlikely to associate with, employ, and work with people affected by depression [25]. In Hong Kong, a study on media reporting of homicides committed by patients with schizophrenia found an increase in negative attitudes and the belief that people with psychosis are dangerous [66]. In the latter month, the online news focused on antigovernment demonstrations regarding the extradition bill. A sequence of protests and marches transpired in opposition to the Hong Kong government’s ratification of the bill, which would allow the transfer of criminal suspects to China, China Taiwan, and China Macau, raising local people’s distress about the potential erosion of autonomy [67]. This event marked the largest protest activities in Hong Kong’s history, as evidenced by a survey conducted from June to August 2019, where >90% of protesters acknowledged that if the government persisted in advancing the bill, protest activities would likely escalate [5,67]. Meanwhile, the rate of depression among locals continued to rise, along with suicidal ideations, surpassing the levels recorded during the larger-scale protests of 2014 [68].

The third leading subtheme centers on the global recession resulting from the economic hardship induced by widespread COVID-19 across industries but with more contradicting positive sentiment. Distinctly, the news story’s structure would initially depict economic challenges and then emphasize the initiative underway to facilitate recovery in response to the crisis. A significant change in overall sentiment occurred, where up to January 2021, the continued decrease in the quantity of positive news ceased. Subsequently, the aggregate number of positive news exceeded negative news almost every month, signalizing an overall shift from negative to positive sentiment surrounding the discussed economic circumstances. This may be attributed to the fact that people were forced to adapt to a “new normal” and start to restore health, economy, and societies after the pandemic had emerged for one year [69]. Strict social distancing measures were gradually relaxed worldwide, such as school reopening, shortened quarantine days, and global trade resumes [70]. Meanwhile, the Hong Kong government has also launched various initiatives and campaigns to stimulate the economic market through issuing digital consumption vouchers to eligible residents; supporting enterprises and the general public with tax cuts; and subsidizing older adults, working families, and child-raising families with more allowances [71].

The fourth and sixth subthemes share greater similarities, as observed on the MDS graph. The fourth subtheme, lifestyle, presented a more positive sentiment by offering different healthy lifestyle suggestions and ways to help its readers improve their well-being. This can be credited to the insights gained from the analysis of coping strategies. Individual adjustment emerged as the second most prominent coping strategy category, emphasizing ways to mitigate depression by considering altering their lifestyle to promote better health through adjustments, such as maintaining adequate sleep, doing regular exercise, and having a nutritious diet while quitting unhealthy behaviors, such as drinking, smoking, and staying up all night. In recent years, lifestyle-based mental health interventions were suggested to be implemented in clinical practices for adults with depression, with growing recommendations on practicing physical activity and exercise, relaxation techniques, work-directed interventions, sleep, and mindfulness-based therapies [72]. Likewise, the sixth subtheme, health issues, was more inclined toward negative sentiment and shared information about the symptoms and negative effects of mental illness as well as available treatments or therapies that are effective in tackling mental health conditions.

The final 2 subthemes were inclined toward positive and negative sentiment, respectively, focusing on leisure and US politics. The leisure subtheme shared depression-related dramas or narratives about celebrities who were and are experiencing depression. However, it is worth noticing that while reading the news, successful stories of how individuals combat depression are rare. Earlier research found that only 7% of depression-related news covered successful treatment or stories of individuals recovering from mental illness, and only 18% emphasized recovery and rehabilitation of those affected as part of their content [16,29]. The concluding subtheme centers on US politics, specifically emphasizing the rivalry and electoral process for the presidency as well as politicians from different parties who experienced depression.

Finally, based on the coping strategy analysis, we found that information on skills and resources was the leading coping strategy category to offer mental health support for populations with depression. Under this category, the suicide intention and prevention helplines were the most frequently cited information, as depression is a strong indicator of suicide [73]. In contrast, various mental health programs were introduced by local mental health organizations such as Mind HK, Kely Support Group, and the Mental Health Association of Hong Kong to offer counseling services and therapies for people with depression. Under the individual adjustment category, exercise is the most cited one, with leading recommendations on different types of running, yoga, and mindfulness activities to enhance self-efficacy in dealing with depression. Regular exercise can bring positive mood change and serve as an antidepressant for individuals with depression [74,75]. Regarding the emotional support category, friends, family, and loved ones are protective factors [76]. However, having a bad relationship may further exacerbate depressive symptoms [77]. In addition, companion pets played a critical role in reducing feelings of depression, particularly during the COVID-19 pandemic period with poor social contact [78]. Although there exists a variety of coping strategies, the overall quantity of strategies offered in the news is deemed rare. In addition, we have observed the inadequacy of policy interventions or support as well as financial assistance for individuals with depression, which implies the deficiency in the societal level of coping strategies in the news coverage. The news media should raise awareness by incorporating more varieties of coping strategies in depression-related news to leverage its positive impact, which might potentially foster positive mental outcomes among individuals with depression and facilitate early mental intervention [79,80].

### Implications for Future Practices

The media portrayal of depression has a dual effect on its readers. In a positive way, the news can help to reduce the uncertainty experienced by an individual with depression, spread information and knowledge about depression, and increase the recognition and understanding of the public [39,81]. In contrast, news can also exacerbate the social stigma and self-stigma of individuals with depression, resulting in decreasing support resources or information from the public, which may deteriorate the well-being of individuals with depression, and may lead to less intentions of help-seeking [22,32,35]. The subthemes highlighted in the news were predominantly negative, suggesting that Hong Kong news agencies and journalists should expand the range of topics they cover when portraying depression. Preferably, incorporating successful treatment of depression and achievements of individuals with depression as potential subthemes can help to reduce the stigmatization of people with depression and foster a better understanding, positive attitude, and accurate beliefs toward populations with depression among the public [14,24,30]. Meanwhile, the overall sentiment of the news was slightly more positive, with 50.51% (1230/2435) positive versus 49.07% (1195/2435) negative news, suggesting that more efforts are needed to report positive content, which could help reduce the stigmatization toward depression [29]. Containing more coping strategies in the news is also suggestive. Specifically, enhancing the inclusion of individual adjustment category in depression-related news was relatively more achievable and preferable as studies found that improving self-efficiency, a key component of the coping appraisal in the protection motivation theory, is more effective in forecasting health-related intention and behavior than eliciting fear or threat [12,18].

### Implications for Future Research

On the basis of the methods and findings of this research, we would like to suggest several points for interested scholars to expand on the current research. First, we would highlight a potential future research direction focusing on coping strategies. In this study, we have analyzed the coping strategies in the online depression news; however, we do not know whether the public or individuals with depression will literally increase their knowledge and awareness of depression and facilitate health-seeking behaviors among populations with depression. Future research is recommended to explore the social media platforms of these news websites and examine the interactions between the news posts and users’ engagement (ie, comments and likes) to see whether their interactions can promote attitudinal changes and potential links with behavioral change.

Second, from a methodological standpoint, sentiment analysis is a powerful tool, notably through techniques such as EmoBank, which analyzes emotions from both the reader’s and writer’s perspective and examines emotion dimensions, such as valence, arousal, and dominance [82,83]. This technique is constructive to explore the topic proposed earlier. Other more advanced techniques such as the multidimensional relation model (examining the relationships between valence, arousal, and dominance) and tree-based regional convolutional neural network–long short term memory (examining text sequential tendency, text regional information, and even nontext elements such as images in the news) are also encouraged for more in-depth research analysis [84,85]. In contrast, for acknowledgment, different score ranges of sentiment analysis are also available beyond –1 to 1. For example, the Semantic Orientation Calculator and AFINN lexicon-based resources provided a score range from –5 (most negative) to +5 (most positive) [86,87]. The Extended Affective Norms for English Words and Chinese EmoBank range from 1 to 9 (negative-neutral-positive), which may also provide a delicate interpretation and quantification of sentiment analysis results [83,88]. Meanwhile, it is also suggested to perform error analysis in sentiment analysis, as demonstrated in these 2 case studies, whether through different computational support or human inspection [89,90].

Third, we would also encourage the replication of this study design and its application to different mental health problems or language cases. However, it is important to note that the resources required for performing LDA and sentiment analysis may differ. For example, except for alphabetical languages such as English, nonalphabetic languages such as Chinese require other packages and resources such as Jieba and Chinese EmoBank due to differences in the language system [61,83].

### Limitations

The study has several limitations that should be acknowledged and recommended for future research to address and advance. Due to the international nature of Hong Kong society, we primarily selected English-reported news as our focus. English news tends to reach more diverse audiences, including disadvantaged groups such as ethnic minorities and foreign domestic helpers who may not be proficient in Chinese, face more stigmas, and are more susceptible to mental health issues [91-93]. However, for future research, this study has presented the steps for investigating the news reports of a mental health issue. Future research can also consider incorporating Chinese as another mainstream language to provide a more comprehensive understanding of portraying depression in Hong Kong, particularly focusing on the local population.

Second, after news coverage collection, we recognized the practical flaw of the imbalance of the number of news items being collected from *SCMP* and *HKFP*. This imbalance may be primarily due to *SCMP* standing out as the most widely read paid newspaper in Hong Kong in terms of news and current affairs, according to the Hong Kong Media Survey report [94]. In contrast, the *SCMP* website places special emphasis on mental health and has dedicated a section called the “Wellbeing” page to report on mental health issues, including depression, which may contribute to the imbalance in coverage between these two news websites.

Third, while screening the news coverage, a further step can be implemented in the screening process, which is to exclude news stories that mention depression as only an incidental topic rather than as the main topic of the news coverage [42]. Future research could explore the possibility of excluding news articles that mention “depressed” or “depression” only once, whether through NLP techniques or human inspection, to ensure a more focused analysis targeting mental health–centered discussion.

### Conclusions

In conclusion, this study is the first to explore latent subthemes, sentiment, and coping strategies in online depression-related news in Hong Kong based on available research. The findings offer a deeper understanding of the underlying subthemes, revealing a predominance of slightly more positive sentiments, inadequate coping strategies, and associated categories that warrant increased attention. For future news reporting, it is recommended that news organizations and journalists take into account the recommendations discussed in the Implications for Future Practices section. Future research is also recommended to explore the topics and methodologies discussed in the Implications for Future Research section and tackle the acknowledged limitations. We encourage the application of NLP techniques to other prevalent mental health disorders such as anxiety, schizophrenia, and bipolar disorder and in different language contexts. In general, this study enhances our comprehension of how depression is portrayed in online news coverage in Hong Kong, particularly during and after periods of social unrest and the COVID-19 pandemic, by providing the latest findings and perspectives.

## Abbreviations

HKFP: Hong Kong Free Press
LDA: latent Dirichlet allocation
MDS: multidimensional scaling
NLP: natural language processing
NLTK: Natural Language Toolkit
RQ: research question
SCMP: South China Morning Post
VADER: Valence Aware Dictionary and Sentiment Reasoner

## Data Availability

The datasets generated and analyzed during this study are available from the first author on reasonable request.
